# Beyond clinical engagement: a pragmatic model for quality improvement interventions, aligning clinical and managerial priorities

**DOI:** 10.1136/bmjqs-2015-004453

**Published:** 2015-12-08

**Authors:** Samuel Pannick, Nick Sevdalis, Thanos Athanasiou

**Affiliations:** 1 NIHR Imperial Patient Safety Translational Research Centre, Imperial College London, London, UK; 2 Centre for Implementation Science, King's College London, London, UK; 3 Department of Surgery & Cancer, Imperial College London, London, UK

**Keywords:** Healthcare quality improvement, Implementation science, Management, Quality improvement methodologies

## Abstract

Despite taking advantage of established learning from other industries, quality improvement initiatives in healthcare may struggle to outperform secular trends. The reasons for this are rarely explored in detail, and are often attributed merely to difficulties in engaging clinicians in quality improvement work. In a narrative review of the literature, we argue that this focus on clinicians, at the relative expense of managerial staff, has proven counterproductive. Clinical engagement is not a universal challenge; moreover, there is evidence that managers—particularly middle managers—also have a role to play in quality improvement. Yet managerial participation in quality improvement interventions is often assumed, rather than proven. We identify specific factors that influence the coordination of front-line staff and managers in quality improvement, and integrate these factors into a novel model: the model of alignment. We use this model to explore the implementation of an interdisciplinary intervention in a recent trial, describing different participation incentives and barriers for different staff groups. The extent to which clinical and managerial interests align may be an important determinant of the ultimate success of quality improvement interventions.

## Introduction

Over the last decade there have been considerable efforts to evaluate and improve the quality of healthcare delivery. Three to six per cent of inpatient deaths may be preventable,^1–4^ and early attempts to foster better care invoked crew resource management in aviation,^5^ industrial quality assurance techniques^6^ and transparent outcome reporting.^7^ However, the pace of improvement remains sluggish,^8^ despite an awareness of how infrequently patients receive the best therapy already available.^9^ ^10^

Quality improvement (QI) interventions have enormous potential to improve healthcare delivery, but well-publicised research successes have proved difficult to replicate outside the trial setting.^11–17^ Discrete QI interventions also struggle to outperform the secular trend towards system-wide improvement.^17^ ^18^ The challenges of reproducing QI successes on a wider scale remain poorly understood, but two key factors are often cited: the engagement of clinical staff in the broader initiative, and the context in which it takes place. Neither clinical engagement^19^ nor context^20^ is well defined, yet the two have become de facto explanations for QI failure. Moreover, the numerous attempts to explain how context affects QI^21–25^ risk overwhelming researchers and clinicians,^21^ with limited ‘how-to’ support for those implementing change.^20^ Here, we discuss whether the focus on clinical staff is misplaced, and propose a novel, pragmatic model for the development and selection of effective, durable QI interventions.

In a narrative synthesis guided by insights from a recent trial (see box 1), we first explore the varying definitions of clinical engagement. We go on to discuss the specific challenges of clinical engagement in QI, and strategies shown to circumvent them. Next, we explore the role of managerial staff, whose importance in QI implementation has been underestimated. Bringing these concepts together, we outline a ‘model for alignment’, highlighting key factors of practical importance for successful QI. The model is then used to retrospectively describe the implementation of an interdisciplinary intervention (see box 2). The model for alignment emphasises that QI efforts must explicitly meet aligned clinical, managerial and organisational needs if they are to become ‘business as usual’.

Box 1 **Autoethnographic observations from the Hospital Event Analysis Describing Significant Unanticipated Problems (HEADS-UP) study^83^**

- The HEADS-UP study evaluated an interdisciplinary team intervention tailored for general medical wards: a structured, daily safety and quality briefing.^83^ ^92^
- HEADS-UP briefings were designed to embed proactive organisational risk surveillance into routine ward care. Through structured discussion, clinical staff would identify risks to the delivery of high quality care on a daily basis, addressing them promptly through facilitated communication with senior clinicians and managers, before patient harm occurred.
- One of the authors of this paper (SP) was heavily involved in HEADS-UP implementation at two institutions, and in facilitating the use of the data arising from it—a form of participatory research.^93^ His observations, discussions with staff, reflections on clinical governance proceedings and implementation challenges were recorded in field notes over a 20-month period.
- These ‘autoethnographic’ insights, from researchers embedded within their host organisations, are widely used in organisational case study research, providing rich accounts of culture and practices.^51^ ^94^ ^95^
- Observations from the HEADS-UP study informed the narrative synthesis of the literature described here, and the construction of the model of alignment for successful quality improvement interventions.

Box 2 **Applying the model of alignment to analyse Hospital Event Analysis Describing Significant Unanticipated Problems (HEADS-UP) implementation at one study site**

- Poor engagement with incident reporting was an organisational concern. However, there was little capacity to enact an improvement programme. No other goals were set aside to prioritise the HEADS-UP programme. As an experimental intervention, the efficacy of HEADS-UP and its implementation strategy was unknown. (***Strategic selection of quality improvement (QI) target and intervention***)
- Front-line staff expressed initiative fatigue at the beginning of the study, but the face validity of the HEADS-UP tool (and its co-design) mitigated much of their expected resistance, and HEADS-UP was incorporated into normal workflow. QI participation was not formally rewarded, but some junior clinical staff were able to exploit their involvement in HEADS-UP to help with career progression. Others reflected that they found HEADS-UP useful for their own practice and that it improved the quality of interdisciplinary care for their patients, which may have been perceived as a benefit or reward. (***Incentives and actions for front-line clinical staff***)
- Middle managers were not personally incentivised to participate in HEADS-UP, although an endorsement by the Care Quality Commission during the study period prompted senior managers to designate a greater focus on the programme. Where HEADS-UP generated information to bolster middle managers’ existing business plans and develop new ones, those managers coordinated staff-identified opportunities for service development with linked organisational priorities. They gave HEADS-UP their personal backing, encouraging its use, although no additional resources were available to accompany this support. In contrast, middle managers for whom HEADS-UP was less directly useful did less to hold their service areas accountable for HEADS-UP performance. HEADS-UP piggybacked onto existing staff meetings and governance structures: no protected time was made available for involvement in dedicated training, analysis or feedback. Middle managers’ time and attention was strictly limited, and competing priorities (eg, the implementation of an electronic health record, or a forthcoming merger) often precluded meaningful progress with this QI intervention. Interestingly, resolution of the persistent issues raised in the HEADS-UP briefings appeared to depend less on each one's inherent safety threat, than on agreement between clinical staff and managers of the need for change. (***Incentives and actions for middle managers***)
- The organisation focused heavily on clinical quality, investing in external consultants to help develop new clinical services and improve the efficiency of existing ones. Board members also dedicated substantial time to clinical quality issues. In fact, clinical quality was the focus of numerous committees and subcommittees, with a complex, devolved governance structure. However, the regulatory environment did not allow for a self-determined quality strategy, with quality priorities established largely by the local healthcare commissioning body and a national quality inspectorate. Board-level meetings were awash with clinical quality metrics, among which the ‘softer data’ emerging from the HEADS-UP briefings had a less certain place.^96^ As the organisation moved to merge with another institution, fewer resources were available for continuous QI in the interim. (***Incentives and actions for senior managers***)

## Engagement—a reciprocal commitment from staff and their organisation

There is no universal definition of engagement: it may be an attitude, behaviour, an outcome—or all three.^26^ Schaufeli *et al*^27^ describe engagement as an employee's positive motivational state, characterised by ‘vigour, dedication and absorption’. A broader, more cooperative, position is that engagement is a two-way phenomenon, with an onus on the organisation to establish conditions encouraging engagement and the opportunities for it to be manifest.^28^ Clinical engagement, then, involves staff actively contributing ‘within their normal working roles to maintaining and enhancing the performance of the organisation, which itself recognises this commitment in supporting and encouraging high quality care’.^28^ This working definition makes it clear that real engagement is a very different entity to staff acquiescence, for which it is often confused.^29^ ^30^

In QI, however, clinical engagement has been summarised simply as staff's ‘active involvement’,^31^ with no recognition of the possible dialogue between clinicians and those seeking to improve their performance. That no organisational contribution is expected may go some way to explaining why clinical engagement has been problematic. Although there is specific literature pertaining to physician engagement, in this discussion (unless specified otherwise) we group clinical healthcare professionals together. QI interventions are typically interdisciplinary, and securing greater engagement of a single staff group is not an end in itself, only a step towards an ‘organisational culture where all staff feel valued and involved’.^30^

## Clinical engagement can be improved by co-design, local modification and strategic selection of QI interventions

Healthcare professionals have been reluctant to involve themselves in QI initiatives.^31^ This is especially apparent in periods of sustained organisational turbulence, but is a long-standing, multifactorial and international problem.^31^ Doctors are disproportionately hesitant to participate in safety behaviours like incident reporting,^32^ ^33^ and active resistance from senior staff remains the most common barrier to the successful implementation of interdisciplinary safety checklists.^34–38^ The narrative of ‘automatic’ clinical resistance to new initiatives, or ‘change fatigue’,^31^ is seemingly widely accepted within the QI literature. Circumventing this fatigue is considered a major triumph, even fundamental to QI success. With few exceptions,^24^ ^25^ local QI breakthroughs are attributed to good clinical engagement; conversely, failures are seen only through the prism of inadequate clinical buy-in.

Not all interventions are subject to the same clinical disengagement barrier, however: some programmes might have lower thresholds for participation.^21^ ^39^ ^40^ Conversely, problems with the introduction of a specific improvement strategy do not necessarily indicate a wider reluctance to change practice. The intervention's characteristics, at least in part, determine its reception. In our experience, iterative co-design of a structured quality and safety briefing intervention with physicians, to maximise its face validity, mitigated much of their expected resistance (see box 2). The process of co-design may also in itself improve staff ownership of the intervention. Other strategies may also have immediate appeal: interventions that used peer facilitation,^41^ wider reporting options and feedback,^42–47^ or engaging whole teams to identify problems^48–50^ all seemingly fell on fertile ground.

Even when QI strategies do not appeal intuitively to clinicians, generating clinical engagement need not prove an insurmountable challenge. Defining the ‘soft periphery’ of a QI programme—the elements that should be flexibly adapted to optimise the programme's acceptance, without invalidating the entire intervention—is key.^51^ Making the effort to appropriately modify QI tools for the context in which they will be applied (eg, creating separate versions of surgical safety checklists for different specialties) then makes those tools much more palatable for clinicians.^34^ In fact, local adaptation is the *most* commonly cited factor affecting checklist implementation, more so even than resistance from specific clinicians.^34^ This reflects each organisation's responsibility to create the opportunities for meaningful engagement: active clinical involvement is more likely when QI tools have been purposefully tailored, and when there is protected time for training to use them.^31^ ^34^ In contrast, unmodified checklists are unlikely to be used as intended, nor improve patient outcomes—regardless of hospitals’ reported compliance.^13^ ^52^

While co-design and local modification do improve the adoption of QI interventions, the financial and opportunity costs of pre-existing efforts represent a major challenge to any new initiative. Relentless organisational change, with little sense of an overall strategic direction, also contributes to a general ennui.^31^ Clinicians, believing that each ‘fad’ will soon be replaced with another, feel there is ‘little point in investing heavily in any one initiative’.^31^ The strategic selection of a limited number of QI interventions, appropriate to the organisation's capacity to implement them, is therefore crucial.^23^ ^53^ Experts have identified 22 patient safety strategies with a sufficient evidence base to recommend their widespread adoption:^54^ ^55^ organisations may choose to focus on these first, with a view to their specific local needs. However, if organisations are to select only the QI targets that they have the capacity and willpower to pursue, other potentially valuable initiatives need be deferred in the interests of preserving engagement and momentum. Regulatory bodies have an important role here: they should give institutions the time and space to develop these focused improvement strategies.^56^ It may seem odd to decry the slow pace of improvement, and yet advocate a more deliberate, institution-specific approach. With space for self-determination, however, organisations that strategically shape their QI attempts go on to see wider benefits, tackling deep cultural issues that go unaddressed with more haphazard approaches.^56^

## Beyond clinical engagement: the role of managers in QI

Improving clinical engagement is only part of the solution to ineffective QI: ‘administrative engagement is equally important, or disillusionment… ensues’.^29^ Quality of care is not a leading priority for many hospital boards, however.^57–59^ Although board-level attention to quality issues has been associated with clinical quality,^57^ ^58^ ^60^ how this commitment translates, in practice, into front-line action remains unclear.^61^ A recent survey study provided a key insight: board and middle management practices are linked, and correlate strongly with hospital performance on clinical quality metrics.^62^ Certain board characteristics were specifically linked to middle management styles: board attention to quality was associated with management practices that monitored it, and the use of quality metrics at board level corresponded to good operational management and target setting.^62^ If good management is truly associated with clinical quality, the role of managers in QI deserves further attention.

Yet managerial participation in QI interventions is often assumed, rather than analysed in detail. Although senior hospital executives may participate constructively in collaborative safety programmes,^14^ ^63^ more often, active managerial involvement goes no further than the ‘expressions of support’ described in many QI reports. More detailed evaluations describe difficulties recruiting executives to work with QI teams, even as part of major safety initiatives.^25^ When they do engage, managers have a different outlook on quality and safety programmes to clinical staff, perceiving different components of the programme to be valuable and holding more positive views of the overall results.^64^ Meaningful input from managers is important for the design, monitoring and evaluation of QI interventions;^64^ simply obtaining their permission to proceed is not enough.

There is little published work on the role of managers in QI, the majority of which relates only to senior (board-level) managers, rather than the middle managers under their supervision.^59^ ^61^ Importantly, most improvement initiatives fail to specify how they engage these middle managers, with whom front-line staff interact directly and regularly. Middle managers are a particularly heterogeneous group, with diverse professional backgrounds,^61^ often promoted on the basis of a technical skill set rather than any specific leadership or management ability.^65^ Many have ‘hybrid’ clinical and administrative duties,^61^ ^66^ with an inherent tension between those roles;^67^ their decisions are necessarily ‘constrained, contested and political’,^68^ but favour knowledge drawn from experience rather than research findings.^69^ ^70^ Little more is known about the cognitive biases that affect middle managers’ judgements, but enthusiasm for QI is not automatic. For example, they may feel the operating costs of QI programmes are not justified by any potential future benefits.^71^ There remains a pressing need for research into how healthcare managers balance their multiple fiscal, statutory and service responsibilities.^59^

## Influencing middle managers to facilitate effective QI: status, incentives and resources

It appears, then, that managers contribute to organisational quality; that their active involvement in QI has been taken for granted rather than proven; and that their decision making relating to QI is likely to be complex, with conflicting priorities that are not easily resolved. Yet middle managers, in particular, are uniquely placed to facilitate effective QI. They have the power to accelerate or impede the implementation of innovations,^61^ mediating organisational messages for front-line staff, but also upwardly influencing their seniors to draw attention to the high-level support needed for specific QI programmes.^72^ ^73^ Acting as information brokers, translating organisational strategy into actionable tasks, and promoting innovative practice, middle managers can convince clinical staff to prioritise QI implementation among numerous competing demands.^61^

Harnessing middle managers’ ability to broker organisational and front-line attention to a QI programme may prove essential to its success: proactive commitment from middle managers does influence effective QI implementation.^74^ Although many advocate a clinician-led, ‘bottom-up’ approach to improvement,^75^ staff-driven initiatives that do not align well with strategic priorities have only limited impact or longevity.^71^ ^76^ ^77^ Clear tensions emerge when QI efforts are delegated entirely to clinicians without support for their direction and goals.^71^ Without more senior support, front-line staff are unable to marshal the resources required to spread change,^78^ and managers have an important role to play in navigating cross-departmental obstacles.^59^ Managers who effectively facilitate QI, without micromanaging it, are well appreciated by front-line staff.^78^ The subsequent pace of change may be slow, but a combination of top-down and bottom-up implementation results in a lasting impact.^78^

Senior managers play a role in determining middle managers’ commitment to QI. These senior managers should directly emphasise QI as an organisational priority, incentivise QI commitment in performance reviews, and—vitally—make available the necessary resources.^72^ In addition, encouraging middle managers to leverage the human resources and performance reviews at their own disposal also improves their commitment to QI implementation.^72^ Interestingly, a performance-related human resources management framework for clinicians has recently been described and implemented, encountering little of the expected physician resistance.^79^ Transparent negotiation of QI goals at each organisational level may therefore be feasible and necessary for high quality implementation.

## A model of alignment for successful QI

We suggest that neither clinicians nor managers can make meaningful QI progress in isolation: their collaboration is fundamental to sustainably embedding practice innovations. The choice of a QI intervention, and its implementation model, both need coordination between clinical and managerial teams. We have discussed, in the preceding sections, how each group might be motivated to take part in this process. Yet their efforts need to be aligned, if QI is to form a significant part of their workload, and not be overwhelmed by other priorities.^62^ We highlight some examples of QI programmes in which the degree of collaboration between front-line and managerial staff may have contributed to the ultimate outcome (table 1). In trials reporting significant improvements, investigators ensured there was adequate managerial participation—or took on managerial roles themselves—to complement clinical involvement.^11^ ^12^ ^15^ ^80^ Similarly, implementation and spread of a QI intervention in a real-world setting was best accomplished with the co-leadership of top-level administrators and front-line champions.^78^ Where managerial engagement was lacking, interventions did not improve outcomes significantly, or systems defects did not prove amenable to the efforts of clinical teams alone.^13^ ^17^ ^81^

**Table 1:** Descriptions of managerial collaboration in selected quality improvement (QI) interventions

QI intervention	Implementation phase (proof of concept/trial/scaling up)	Managerial collaboration	Outcome
Surgical safety checklist	Trial^11^ ^12^ ^80^	Systems changes facilitated by the local investigator—essentially fulfilling a dedicated managerial role.^11^ Hospital administration/management leaders required to ‘support the intervention’^11^ ^80^	Reduced in-hospital complications
	Scaling up^13^	No assessment of managerial involvement in mandatory checklist implementation. Meaningful local implementation unlikely to have taken place^52^	No significant change in patient outcomes
Program to reduce central line infections	Trial^15^	Program targeted middle managers and senior hospital leaders as well as front-line staff.^24^ Chief executives wrote ‘commitment letter’ to the program team. Nurse manager led the project locally; project team also included a hospital executive advocate	Reduced infection rates
	Scaling up^17^	Chief executives agreed organisations would participate, and that a director would join the local project team. In practice, most units struggled to involve executives^25^	No improvement compared with controls
Program to detect and mitigate organisational weaknesses	Proof of concept^81^	Executive sponsor for each site team. Managerial staff less often directly involved as project team members	System defects not tractable to small clinical teams’ QI methodology
Program to improve interprofessional coordination	Scaling up^78^	Spectrum of managerial involvement. In ‘bottom-up’ hospitals, administrators delegated and served as resources. In ‘top-down’ hospitals, managers primarily drove the change effort	Co-leadership of top-level administrators and front-line champions best facilitated implementation and spread of the intervention

**Figure 1:**
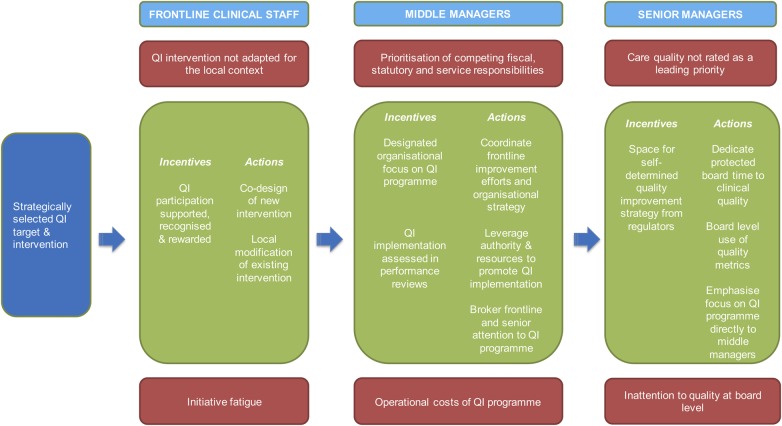
The model of alignment. Strategically selected quality improvement targets and interventions successfully align the interests of clinicians and non-clinicians at multiple levels within the organisation. At each level, staff engagement with these interventions is facilitated by deliberate incentives to prioritise it, the recognition of competing priorities and barriers to involvement, and actions to address them.

With this in mind, we propose a simplified model for successful QI interventions (figure 1). This model emphasises, foremost, that QI interventions aiming to change healthcare providers’ practice should aim to meet the aligned needs of staff at multiple levels in the organisation. Failing to coordinate these interests renders interventions susceptible to failure, regardless of enthusiasm and engagement at the other organisational levels. In fact, the degree to which an intervention recognises, makes use of, or conflicts with existing staff priorities is fundamental to its success, and should not be considered in the accompanying implementation strategy only. This pre-emptive consideration of where an intervention is likely to garner support, and the conflicts that need to be resolved to allow wholehearted participation, reflect the ‘practical wisdom’ thought to be a critical element of successful QI.^82^ Throughout this narrative literature review, and reinforced by our recent practical experience (box 1), we identified specific facilitators that coordinate clinical, middle management and senior management participation in QI. To build a useful model, we then separated these factors into incentives (establishing each group's QI participation as a core expectation of their work) and actions (specific actions by that group that make QI implementation more effective). We also highlight important barriers to aligned QI, again identified from the narrative synthesis. For facilitators and barriers, we focused deliberately on modifiable factors, with a view to building a valid model that has immediate application in practice. Importantly, the inclusion of managers as core members of the QI team may augment what is actually ‘modifiable’: changes that remain frustratingly out of reach for clinical QI teams^81^ may fall within the remit of an expanded clinical-managerial group.

How could this model be used in practice? Prospectively, clinicians and managers jointly establish and prioritise the challenges facing their service. Multidisciplinary tools systematically collect data relevant to front-line care delivery problems (from staff and patients).^83^ ^84^ Teams then assess and rank the apparent safety threats, for example with streamlined versions of tools like Healthcare Failure Modes and Effects Analysis or Hierarchical Task Analysis.^81^ ^85^ These tools inform the strategic selection of high priority targets for local improvement efforts.

Interventions are co-designed or adapted with clinicians, with a focus on the quality strategies for which effectiveness and implementation evidence is strongest. Clinicians’ participation is supported, recognised and rewarded, perhaps as part of a formal performance management process. Middle managers coordinate alignment of the improvement efforts with organisational goals, and are themselves heavily incentivised to see QI facilitation as a core role of their own. Influencing their supervisors to attract organisational support and resources for QI efforts, middle managers’ interest in quality is further reinforced by protected board time for quality issues, and board-level use of quality metrics. Board members may need to robustly engage with other stakeholders in the local healthcare economy to generate (and protect) an institution-specific quality strategy.

The model can also be used retrospectively, to describe how interventions were implemented in practice, and to explain their effects. We use the model to explore a recent interdisciplinary intervention in our own institutions, describing mutable influences on staff over the course of a trial (see box 2). Definitive progress on a number of key issues occurred only when front-line staff and middle managers agreed the need for change.

We feel the proposed model of alignment is a useful, novel concept for a number of reasons. First, it emphasises the need to go beyond clinical engagement. Second, it highlights the role of non-clinical staff in sustaining effective QI. Most importantly, it integrates separate literature streams on clinical and managerial influences on QI, lending itself to the prospective design of interventions as well as the retrospective analysis of why they achieved their goals or not. Interestingly, multilevel interventions (explicitly addressing patient, professional and organisational factors, for example) show the most consistent improvements in process and clinical outcomes.^86^ The model of alignment suggests how we might incorporate a similarly multifaceted approach into the design of any new QI intervention. Other authors have recently raised concerns about ‘colliding’ QI interventions, conceived in isolation but effectively competing in a limited marketplace.^87^ Our model encourages a broader analysis of the environment into which any new QI intervention is launched.

We hope this model will prove useful for future interventions, which will need to more explicitly link their assumptions with underlying theory.^88^ However, it does not negate the value of existing models, which remain well placed to structure QI reporting and evaluation.^21–23^ The model of alignment was derived from a narrative review of the literature, drawing on prior systematic reviews,^31^ ^59^ and tested retrospectively against the experience of implementing a single intervention. It requires more empirical validation, particularly in the prospective design of novel interventions. Lastly, though parsimony is necessary for a ‘good’ theory of context,^88^ the model of alignment may be too simplistic for some analyses.

## Conclusion

Critics of implementation research argue that its theories are no more helpful than common sense.^89^ ^90^ Proponents reply that these theories are open to questioning, while common sense—in itself an informal, ‘lay’ theory—relies on implicit assumptions that are difficult to challenge.^20^ What we have described here, we hope, might satisfy both camps. Sharing the concern that existing theories offer little to practitioners at the sharp end, and informed by our own recent experience of a complex QI intervention, we offer a focal point for the design and evaluation of future attempts to improve healthcare delivery. The extent to which QI aligns the interests of front-line staff and their managers has not previously been explored in this way.

Perhaps, in truth, we have historically expected too much of clinicians in QI, and demanded too little of their managers. Previous assumptions that managers are well prepared to make meaningful contributions to QI interventions have not been substantiated.^59^ The next generation of guidelines for QI reporting will emphasise narrative understanding,^91^ and this should extend to fuller descriptions of whether clinical and managerial priorities coincided (or collided) within the context of the intervention. Although there are few hard barriers to either group's participation in QI, competing demands force clinicians and managers to rationalise their efforts, and in some cases consciously relinquish other priorities. Developing effective and sustainable QI interventions may depend on our ability to align the two groups’ divergent interests.
